# Supplementation of polyclonal antibodies, developed against epitope-string toxin-specific peptide immunogens, to commercial polyvalent antivenom, shows improved neutralization of Indian Big Four and *Naja kaouthia* snake venoms

**DOI:** 10.1016/j.toxcx.2024.100210

**Published:** 2024-09-26

**Authors:** Abhishek Chanda, Nitin C. Salvi, Pravin V. Shelke, Bhargab Kalita, Aparup Patra, Upasana Puzari, Milind V. Khadilkar, Ashis K. Mukherjee

**Affiliations:** a Microbial Biotechnology and Protein Research Laboratory, Department of Molecular Biology and Biotechnology, School of Sciences, Tezpur University, Tezpur, 784028, Assam, India; b Department of Pharmaceutical Sciences, College of Pharmacy, Oregon State University, Corvallis, 97331, Oregon, USA; c Premium Serums and Vaccines Pvt. Ltd, Narayangaon, Pune, 410504, Maharashtra, India; d Amrita Research Centre, Amrita Vishwa Vidyapeetham, Faridabad, Haryana, 121002, India; e Division of Life Sciences, Institute of Advanced Study in Science and Technology, Vigyan Path Garchuk, Paschim Boragaon, Guwahati, 781035, Assam, India

**Keywords:** Big Four snake venom, Indian monocled cobra, Venom-antivenom interaction, Improved treatment of snakebite

## Abstract

Snakebites profoundly impact the rural population of tropical nations, leading to significant socio-economic repercussions. Polyvalent antivenom (PAV) therapy faces several limitations, including intra-specific variations and poor efficacy against some major toxins and low molecular mass, poorly immunogenic toxins, which contribute to increased mortality and morbidity rates. Innovative strategies for developing novel antivenoms are continuously explored to address these challenges. The present study focuses on designing of 17 epitope-string toxin-specific peptide immunogens from pharmacologically active major and/or poorly immunogenic toxins (snake venom metalloprotease, Kunitz-type serine protease inhibitor, phospholipase A_2_, three-finger toxin) from the venom of the ‘Big Four’ venomous snakes and *Naja kaouthia* (NK) in India. These custom peptide antibodies demonstrated robust immuno-reactivity against the venoms ‘Big Four’ and NK. When these antibodies were supplemented with commercial PAV at a defined ratio (formulated polyvalent antivenom or FPAV), it significantly enhanced the neutralization of snake venom enzymes and *in vivo* neutralization of lethality and pharmacological activities such as haemorrhage, necrosis, pro-coagulant, defibrinogenation, and myotoxicity of ‘Big Four’ and NK venoms compared to PAV in mice. The present study highlights a promising strategy for developing next-generation antivenoms using synthetic peptide-based immunogens, offering a targeted approach to address the limitations of current antivenom therapy.

## Highlights

• Snakebites have a substantial socio-economic impact on rural people in tropical countries.
• Toxin-specific peptide immunogens were designed from ‘Big Four’ and *Naja kaouthia* venoms.
• The custom peptides antibodies were supplemented with commercial Indian polyvalent antivenom.
• Formulated antivenom improved the neutralization of lethality and venom pharmacological effects.
• The work highlights a viable strategy for peptide-based immunogens to create next-gen antivenoms.

## Graphical abstract

**Figure:**
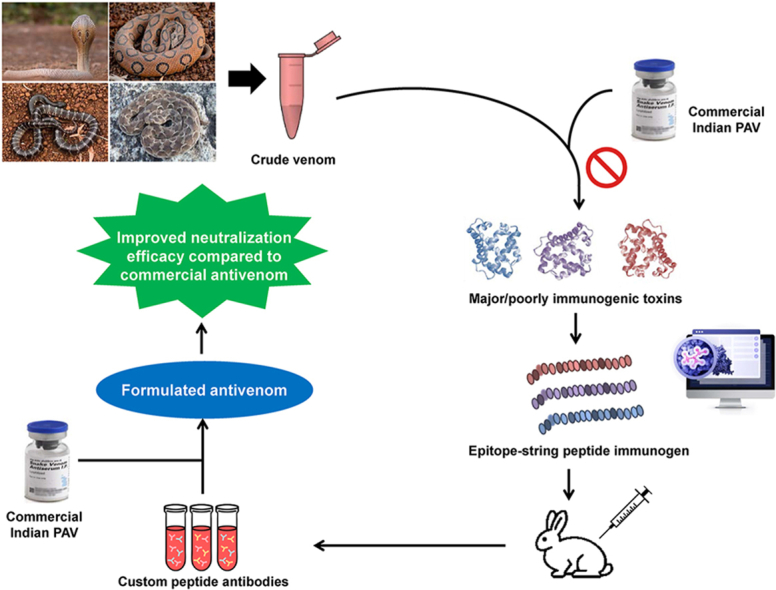

## 1 Introduction

Snake envenomation is one of the most neglected tropical diseases, primarily affecting the rural populations of tropical and subtropical regions of Asia, Africa, and South America (Kasturiratne et al., 2008; WHO, 2019). The annual estimates indicate approximately 2.7 million snakebite cases worldwide, of which ∼140,000 cases are fatal (Gutiérrez et al., 2017; WHO, 2019). India encounters about 1.1–1.7 million cases of snake envenomation annually, among which 58,000 instances are fatal (Mukherjee et al., 2020; Suraweera et al., 2020). The primary victims of this occupational hazard are the poor agricultural workers, laborers, and shepherds, often the chief breadwinners of their families, causing severe socio-economic repercussions to these vulnerable segments of the population (Bawaskar et al., 2017; Gutiérrez et al., 2017). Therefore, snake envenomation is a severe socio-economic challenge in many parts of the world rather than just a public health issue.

The anti-snake venom therapy remains the solitary, established option for treating snakebite victims. In India, the polyvalent antivenoms (PAV) are manufactured against the ‘Big Four’ snake venoms (Russell's viper, *Daboia russelii*; Saw-scaled Viper, *Echis carinatus*; Spectacled Cobra, *Naja naja*; and Common Krait, *Bungarus caeruleus*). The venoms are primarily obtained from Southern India and show less efficiency in neutralizing the venoms from other regions (Kalita et al., 2018a; Warrell et al., 2013). PAV therapy, consisting of polyclonal antibodies or antibody fragments derived from the plasma of hyperimmunized animals, has saved countless lives; however, it shows some adverse effects (Alirol et al., 2017; Deshpande et al., 2013; León et al., 2018; Mukherjee et al., 2020). Moreover, intra and inter-specific variations in the snake venom due to various factors (bio-geographic distribution, sex- and age-based, seasonal) lead to the development of distinct clinical outcomes, severely hampering the PAV efficacy.

Due to the lack of *N. kaouthia* venom-specific antibodies, the PAV in India shows less potency in neutralizing the toxicity of this venom (Chanda et al., 2018; Deka et al., 2019; Kakati et al., 2022; Rashmi et al., 2021). Furthermore, *in vitro* assessments have reported the poor effectiveness of commercial antivenoms in the immunorecognition of low-molecular-mass (<20 kDa) snake venom toxins (Chanda et al., 2019; Kalita et al., 2018c; Patra et al., 2019; Sintiprungrat et al., 2016; Tan et al., 2015a). Additionally, the amount of therapeutically cognate antibodies may be as low as 10–15% in each antivenom vial (Casewell et al., 2010; Patra et al., 2021a; Pla et al., 2019), resulting in the usage of a large amount of antivenom for an effective treatment. The above reasons consecutively surge the charge of treatment, and the infusion of such vast amounts of redundant antibodies further leads to fatal complications.

These shortcomings of the accepted antivenom therapy have marshalled the development of several innovative strategies to explore and develop next-generation antivenoms with enhanced specificity and efficacy. Some of these strategies include the use of medically relevant immunogens (synthetic peptides, recombinant toxins, or epitope DNA strings) to immunize the animals instead of the whole venom (Azofeifa-Cordero et al., 2008; Liu et al., 2021; Wagstaff and Harrison, 2006); employing humanized monoclonal antibodies to neutralize the venom toxins (Khalek et al., 2024; Laustsen et al., 2017; Roncolato et al., 2013); utilize the natural toxin-neutralizing proteins from different animal species (Biardi et al., 2006; Lizano et al., 1997; Neves-Ferreira et al., 1997; Trento et al., 2001); use of natural or synthetic venom toxin inhibitors (Howes et al., 2007; Lewin et al., 2016; Puzari et al., 2021), and use of nanotechnology for targeted delivery of highly stable toxin-neutralizing nanoparticles (Karain et al., 2016; O'Brien et al., 2018).

Developments in biotechnology and bioengineering have resulted in the increased popularity of synthesizing and applying peptides for various research purposes (Ucar et al., 2019). Peptide antigens are often used to generate polyclonal IgG antibodies in either goat or rabbit that target unique epitopes. Generally, the production of high-quality antipeptide antibodies depends on peptide sequence selection, successful peptide synthesis, peptide-carrier protein conjugation, host animal humoral immune response, the adjuvant used, peptide dose administered, route of injection, and lastly, purification of the antibody (Lee et al., 2016).

In the present study, we have designed 19 synthetic custom peptides targeted against the active (antigenic) sites of the poorly immunogenic major toxins (against which antibodies are poorly produced in horses) of the ‘Big Four’ venomous snakes, and *Naja kaouthia* venom from India. Polyclonal antibodies were raised against the 17 custom peptides in rabbits. The purified custom peptide antibodies (CPA) were pooled and supplemented to commercial PAV at different ratios to perform the venom neutralization study in experimental animals to assess the efficacy of the newly formulated antivenom.

## 2 Materials and methods

### 2.1 Materials

Pooled venom samples of the ‘Big Four’ venomous snakes (*Naja naja*, *Daboia russelii*, *Echis carinatus*, and *Bungarus caeruleus*) from India (obtained from Irula Snake Catcher's Association, Tamil Nadu, India) were kind gifts from Premium Serum and Vaccines Pvt. Ltd., Pune. *Naja kaouthia* venom was obtained from Calcutta Snake Park, Kolkata, India. The commercial polyvalent antivenom (PAV) vials were manufactured by Premium Serum and Vaccines Pvt. Ltd. (PSVPL), Pune, India (Batch No. ASVS (I) ly- 014, expiry date: December 2022). Western blot and ECL substrate reagents were procured from Merck, Germany, and BioRad, USA. All other analytical grade chemicals were obtained from Sigma-Aldrich, USA.

### 2.2 Selection of major and weak immunogenic toxins of ‘Big Four’ snake venoms

The list of predominant venom toxins of the ‘Big Four’ venomous snakes (*N. naja*, *D. russelii*, *E. carinatus*, and *B. caeruleus*) of India and eastern India *N. kaouthia* venom was prepared from the LC-MS/MS analysis data performed and published by different research groups (Ali et al., 2013; Chanda et al., 2018, 2019; Choudhury et al., 2017; Kalita et al., 2017, 2018b, 2018c; Mukherjee et al., 2016; Patra et al., 2017, 2019; Sintiprungrat et al., 2016). Further, the list of venom's partial/negligible immuno-recognized toxins against commercial PAV was prepared from the proteomic-based immune profiling studies of the above venom samples (Chanda et al., 2018, 2019; Kalita et al., 2018b, 2018c; Patra et al., 2017, 2019).

### 2.3 Designing of toxins-epitope targeted custom peptides

The sequences of selected snake venom toxins were retrieved from the NCBI database, and the toxins of the same snake venom protein families were subjected to multiple sequence alignments via the Clustal Omega alignment tool. The EMBOSS antigenic prediction tool determined the antigenic sites of these aligned toxins (https://www.bioinformatics.nl/cgi-bin/emboss/antigenic). The peptides with antigenic propensity greater than 1.0 were selected for the study. Moreover, the active site (catalytic and pharmacological) of enzymatic toxins was determined from the literature survey and UniProt database (https://www.uniprot.org/). Finally, 17 peptide sequences covering the active site/s of the toxin with the highest predicted antigenic score were selected, preferably having a cysteine residue at the C- or N- terminal of the peptide sequence for Keyhole limpet hemocyanin (KLH) conjugation.

### 2.4 Physico-chemical characterization of antigenic peptides

The physical and chemical properties of the antigenic custom peptides, such as molecular mass, solubility, and molar extinction coefficient, were analyzed in Innovagen's Peptide Property Calculator (https://pepcalc.com/). The custom peptides with poor aqueous solubility were redesigned by replacing the hydrophobic amino acid residues with neutral or hydrophilic amino acids so that the antigenic propensity of the custom peptide remained unaltered. The designed peptides against the weak immunogenic and major toxins of the Viperidae family (*D. russelii* and *E. carinatus*) and Elapidae family (*N. naja*, *N*. *kaouthia*, and *B*. *caeruleus*) snake venoms were outsourced to Biotech Desk Pvt. Ltd., Secunderabad, Telangana, India, and S Biochem – India, for peptide synthesis. Subsequently, polyclonal antibodies were raised against these peptides in rabbits (see below).

### 2.5 Raising polyclonal antibodies against synthetic custom peptides

The production of polyclonal custom peptide antibodies in female New Zealand rabbits (*Oryctolagus cuniculus*) was outsourced to Bhat Bio-tech India (P) Limited, Bangalore, India. All animal experiments were complied with the ARRIVE guidelines and approved by ethical requirements of the “Committee for Control and Supervision of Experiments in Animals (CPCSEA)” (BBI/IAEC/January/2019/02). A total of 25 rabbits were used for antibody production.

#### 2.5.1 KLH conjugation of the custom peptides

After filtering and sterilizing the bespoke peptides, they were coupled with a protein carrier called Keyhole limpet hemocyanin (KLH). KLH and Sulfo-SMCC were dissolved in activation buffer (pH 7.4), 0.9 M NaCl, and 0.1 M Na_2_PO_4_. The cross-linker Sulfo-SMCC, which changes lysine residues into sulfhydryl-reactive maleimide groups, activated KLH. After that, gel-filtration chromatography was used to purify the active carrier protein. The conjugated peptides were purified by gel-filtration chromatography after the custom peptides (3 mg/mL, dissolved in DMSO) were conjugated with maleimide-activated KLH in a 1:1 ratio and incubated overnight at 8 °C (Carter, 1994; Puzari et al., 2023).

#### 2.5.2 Immunization of rabbits with KLH-conjugated custom peptides for raising polyclonal antibodies

Pre-immune serum (approximately 2 mL) was collected from New Zealand rabbits. After 24 h, ∼200 μg of each of the 17 conjugated peptides in Freund's complete adjuvant were subcutaneously injected in separate animals for the primary vaccination. After 15 days of observation, the rabbits were given the first booster dosage (100 μg of each conjugated peptide in Freund's incomplete adjuvant). The second booster dosage (in Freund's incomplete adjuvant) was administered on the tenth day following the injection of the first booster dose. Blood was drawn from the test bleed (ear vein) on the tenth day following the twofold booster dosage. The serum was then separated to titer the sample using ELISA.

In Freund's incomplete adjuvant, the third booster dosage was given ten days following the second. Blood was drawn from the test bleed (ear vein) on the tenth day following the third booster dose. The serum was separated and utilized to titer and assess the unique peptide-specificity of the antibodies produced by dot blot analysis and ELISA, respectively (see below).

#### 2.5.3 Determination of peptide-rabbit polyclonal antibody interaction by dot blot and ELISA

For dot-blot analysis, 1 μL of the conjugated peptide (1 mg/mL) was spotted on the nitrocellulose membrane and dried on a flow-through apparatus. After the spots had dried, the membrane was blocked using a blocking buffer (50 mM Tris-HCl, pH 8, 500 mM NaCl, 0.1% BSA, 0.05% Tween 20). 30 μL of diluted (1: 10,000) pre- and immunized serum was applied to the blots. After removing any attached serum, the blots were dried, and 50 μL of Protein A gold conjugate was added to observe the interaction between the antibody and the antigen. The wash buffer was 50 mM Tris-HCl, pH 8, 500 mM NaCl, and 0.05% Tween 20. In the experiment, the BSA served as a negative control.

For the ELISA, 100 μL of 2 μg/mL KLH-conjugated peptides in PBS (200 ng/well) were coated in the wells of 96-well microtiter plates, and the plates were stored at 4 °C for the whole night. 250 μL of 1% BSA in 1X PBS was used to block the wells, and they were then incubated for 1 h at room temperature. After three rounds of washing the wells with wash buffer (1X PBS with 0.05% Tween 20), 100 μL of the diluted serum samples (1:1000) were added to the first well and further serially diluted two-folds from 1:2000 to 1:204800. The wells were then incubated for 30 min at room temperature (∼25 °C).

After three rounds of washing with wash buffer, 100 μL of Protein A-HRP (Zymed, USA) diluted to 1: 60,000 in blocking buffer (made right before use) was added to each well. The wells were then incubated for 30 min at room temperature (∼25 °C). After using wash buffer to clean the wells thrice, 100 μL of TMB/H2O2 substrate solution was added to each well. The wells were left in the dark for 30 min at room temperature (around 25 °C). In a microplate reader, the absorbance was measured at 450 nm for the primary and 630 nm for the reference wavelength.

#### 2.5.4 Purification of the custom peptide-specific antibodies (CPAs) from rabbit hyperimmunized serum

The caprylic acid precipitation method was followed to isolate F(ab')_2_ from hyperimmunized serum (Page and Thorpe, 1996). Briefly, 25–30 mL antisera were diluted with two parts of distilled water, and the pH was adjusted to 5.5 ± 0.3, followed by the addition of pepsin to digest the non-IgG plasma proteins (albumin, globulin, and fibrinogen) and cleavage of the IgG into bivalent F(ab')_2_ fragments. The pepsin concentration was adjusted according to the amount of the IgG present in the reaction mixture (WHO, 2018). The pepsin-digested antisera were then subjected to thermo-coagulation by heating at 56 ± 1 °C for 1 h and, after that, cooled immediately to 25 ± 2 °C. The caprylic acid (8% v/v) was added slowly to the reaction mixture with constant stirring for 4 h at room temperature (∼25 °C). Subsequently, the solution was centrifuged at 10,000 g for 10 min, the pH of the supernatant was neutralized with 0.01 M NaOH to 6.5 ± 0.2, and lyophilized.

Custom peptide-specific antibodies (CPAs) were purified by affinity chromatography. Briefly, the F(ab')_2_ molecules were incubated with the custom peptides bound NHS-activated sepharose column at room temperature for 2 h, and bound custom-peptide specific F(ab')_2_ was eluted with 0.1 M glycine, pH 2.0 and collected in tubes containing equal volumes of 1 M Tris-Cl, pH 9.0, whereas the unbound fraction was discarded. The absorbance of the eluent was determined at 280 nm, and the protein peak was pooled. The immunoreactivity of the purified CPAs (200 ng) against respective custom peptides (100 ng) was assessed by ELISA following the standard protocol of our laboratory (Kalita et al., 2017; Mukherjee et al., 2016).

### 2.6 Determination of the immuno-recognition capacity of the CPA against the snake venom toxins

The immuno-recognition capacity of the CPAs towards the venoms of the ‘Big Four’ snakes from India was determined by immunoblot analysis. Briefly, 100 μg of crude snake venoms were separated by 12.5% SDS-PAGE under reduced conditions, transferred to PVDF membrane (Merck, USA), and the blots were incubated with 5% fat-free skimmed milk overnight at 4 °C to block non-specific bindings. The membrane was washed three times with 1X Tris-buffered saline containing 0.05% tween-20 (TBS-T), and 100 μL of primary antibody (3.0 ng/μL of CPA in 1X TBS) was added and incubated for 60 min at room temperature. Consequently, the membrane was washed thoroughly with TBS-T before adding HRP conjugated anti-rabbit secondary antibody (1: 15,000 dilutions) and incubated for 60 min at room temperature. The blots were developed using an ECL substrate (BioRad, USA), and the images were captured in an imaging system (ChemiDoc XRS+, BioRad, USA).

### 2.7 Supplementation of CPA to commercial PAV and assessment of *in vivo* venom neutralization potency of formulated PAV

#### 2.7.1 Preparation of CPA pool and supplementation of commercial PAV with pooled CPAs

CPAs against peptides of the same toxin families were initially mixed in equal amounts and pooled. After that, Viperidae- and Elapidae-specific CPAs were prepared, as mentioned below.

The relative proportions of major toxin families in the ‘Big Four’ snakes and NK venom have already been reported in various studies (Chanda et al., 2018, 2019; Dutta et al., 2017; Kalita et al., 2017, 2018b, 2018c; Patra et al., 2017, 2019). These proportions created specific CPA pools for the Viperidae and Elapidae snake families. The mean relative proportions of major toxin families from different species and locations were calculated, and based on these ratios, CPAs were pooled separately for Viperidae and Elapidae venoms (Table 1a).

**Table 1a:** Formulation of pooled custom peptide antibodies (CPAs) effective against venoms of “Big Four” snakes.

Snake family	Protein class	Ratio of CPA (protein: protein)
**Viperidae**	**SVMP**: **KSPI**: **PLA**_**2**_	**1: 1.2: 2.5**
**Elapidae**	**PLA**_**2**_ : **3FTx**	**1: 3**

The pooled CPAs were supplemented to commercial polyvalent antivenom (PAV) at ratios of 1:2 to 1:300 (CPA: PAV; protein: protein) based on the dose-finding assays as described in section 2.7.3 (Table 1b). The neutralization potency of CPA supplemented with commercial antivenom and formulated polyvalent antivenom (FPAV) was assessed.

**Table 1b:** The proportion of pooled custom peptide antibodies (CPA) (in mg) added per g of commercial antivenom to form the formulated polyvalent antivenom (FPAV).

Snake Venom	Proportion of PAV and CPA (mg of CPA/g PAV)	Ratio (CPA: PAV)
*Naja naja*	240	1:4
*Daboia russelii*	273	1:3.8
*Bungarus caeruleus*	541	1:2
*Echis carinatus*	117	1:8.6
*Naja kaouthia*	3.43	1:300

#### 2.7.2 In vitro neutralization of enzymatic activities and pharmacological properties of snake venoms

The neutralization of enzymatic activities and pharmacological properties of PAV and FPAV were evaluated by assessing the metalloprotease, phospholipase A_2,_ and pro-coagulant activities for the Viperidae (*D. russelii* and *E. carinatus*) venoms (Mukherjee, 2014; Mukherjee et al., 2016; Patra et al., 2017). In contrast, the Elapid (*N. naja, N. kaouthia,* and *B. caeruleus*) venoms were assessed for phospholipase A_2_ and anticoagulant activities according to previously described protocols (Chanda et al., 2018; Dutta et al., 2017; Kakati et al., 2022; Patra et al., 2019). Briefly, 10 μg of crude venoms were incubated with 100 μg of either CPAs, PAV, or FPAV for 30 min at 37 °C followed by an assay of the respective activities. The activity of crude venom without antivenom/CPA treatment was considered 100% activity, and other values were compared to that.

#### 2.7.3 Neutralization of lethality in mice model

The neutralizing potency of PAV and PAV supplemented with CPA was tested in laboratory-inbred Swiss albino mice (males and females) weighing 18 and 20 g, aged 3–4 weeks. The preclinical study was conducted at PSVPL's animal house facility in Pune. All animal experiments were performed following the WHO recommendations and ARRIVE (Animal Research: Reporting of In Vivo Experiments) standards and the ethical requirements of the “Committee for Control and Supervision of Experiments in Animals (CPCSEA)" (R&D/ASVS-I/ED_50_-M/02/21). Mice were housed in cages in a typical habitat with temperatures ranging from 22 to 25 °C, relative humidity between 50 and 60%, 12-h daylight cycles, and 12–15 air changes per h (Patra et al., 2021b). Dry food pellets (Nutritivsue Life Sciences, Pune) and filtered water were freely available to the mice for consumption.

As stated previously, the LD_50_ values of venoms in mice were determined (Patra et al., 2021b). Mice were injected with 5 LD_50_ doses (challenging dose) of each ‘Big Four’ snake venom and *N. kaouthia* venom pre-incubated in different dilutions of PAV for 30 min at 37 °C. The PAV dilutions that could not protect the experimental rodents against the challenge dose of Big Four snake venoms and NK venom were determined as dose-finding assays and were selected for further experiments. Subsequently, different dilutions of formulated CPA were supplemented with the selected PAV dilution to form FPAV, pre-incubated with the 5 LD_50_ dose of the snake venoms at 37 °C for 30 min, and then injected into the mice (in a volume of 0.5 mL) to assess the change in the survivability percentage. Six mice (n = 6) were selected for each experiment, and the death/survivability was recorded 24 h post-injection (Patra et al., 2021b).

#### 2.7.4 Additional preclinical tests to estimate how well commercial PAV and FPAV neutralize particular venom-induced pharmacological activities

All the procedures for determining the pharmacological effects of venom were adopted from WHO guidelines and our previous study (Patra et al., 2021b; WHO, 2018).

Five groups of mice were briefly anaesthetized, and their shaved skins were injected intradermally with varying doses of venom solutions (50 μL) or normal saline (control). The mice were effectively put to sleep by carbon dioxide asphyxiation 3 h after the treatment. After removing the injected skin region, the hemorrhagic lesion's dimensions were measured in two dimensions using callipers. The average diameter of the hemorrhagic lesion was measured for every venom dose. The minimum hemorrhagic dose (MHD) was then calculated by plotting these values against the corresponding venom doses. The amount of venom needed to cause a skin bleed with a diameter of 10 mm is known as the MHD (Gutiérrez et al., 1985; Patra et al., 2021b; WHO, 2018).

We conducted the same procedure outlined earlier to assess the venom's necrotizing potential, measuring the size of dermonecrotic lesions (Theakston and Reid, 1983; WHO, 2018). The average diameter of these lesions was calculated for each dose of venom, and these values were then plotted against the corresponding venom doses to establish the minimum necrotic dose (MND). The MND is the venom dose necessary to induce skin necrosis with a 5 mm diameter.

The described procedure outlines a method for assessing venom's *in vivo* defibrinogenating activity using mice as experimental subjects. The venom solutions were prepared in graded concentrations mixed with 0.2 mL of normal saline. These solutions were then administered intravenously to groups of five mice, with an additional control group receiving only normal saline injections. Following a 1-h interval post-injection, blood samples were collected from the anaesthetized mice via cardiac puncture and transferred to glass tubes. Visual observation of the blood samples in the tubes was conducted to determine clot formation, discerned by tilting. Clotting indicates the presence of fibrinogen, a vital protein for clot formation. The MDD is defined as the minimum dose of venom that produces incoagulable blood in all mice tested within 1 h of intravenous injection (Theakston, 1986).

To determine the plasma clotting activity of venom samples, human plasma was incubated with varying concentrations of venom dissolved in 0.2 mL of normal saline. Clot formation was observed visually by tilting the tubes. The minimum coagulant dose (MCD) of venom is defined as the smallest amount of venom (μg/mL) that clots a standard citrated solution of human plasma (fibrinogen content 2.8 g/L) under the same conditions (MCD-P) (Gené et al., 1989).

For assaying myotoxic activity, groups of five mice were administered injections of varying doses of venom dissolved in 50 μL of normal saline directly into the right gastrocnemius muscle. A control group received injections of an equivalent volume of normal saline. Blood samples were then collected from the tail tip of anaesthetized mice after a 3-h interval post-injection (Gutiérrez et al., 1995). The serum creatine phosphokinase (CPK) activity levels were assessed using a diagnostic kit (Tulip Diagnostic, India).

The purpose of the neutralization test was to determine if the venom-antivenom combination could counteract the pharmacological effects of the venom in question. A challenge dose of venom (equivalent to five folds of MHD/MND/MCD-P/MDD/MMD, respectively) was incubated with different concentrations of PAV/FPAV at 37 °C for 30 min, and the neutralization of the venom-induced toxicities was determined by comparing the toxicities with the only venom (Patra et al., 2021b).

## 3 Results

### 3.1 List of toxins and the derived custom peptides

**Fig. 1:**
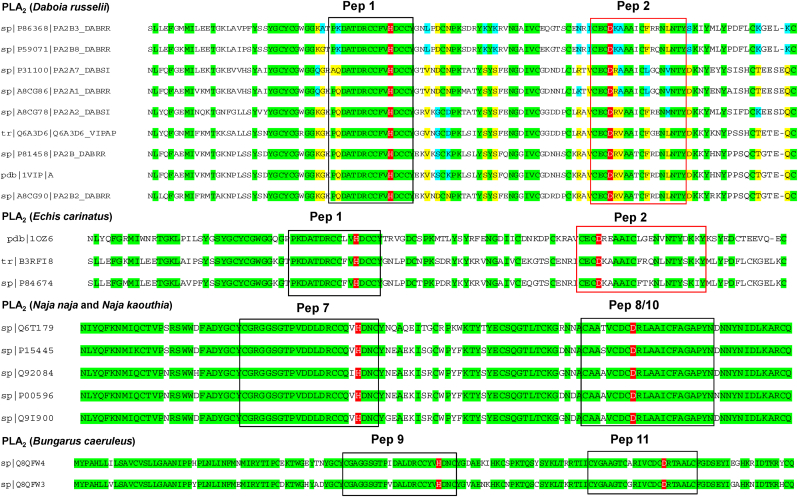
Multiple sequence alignments of phospholipase A_2_ of Viperidae and Elapidae showing the selected peptides (in the box). The conserved regions are marked in green, and the active sites are marked in red. (For interpretation of the references to colour in this figure legend, the reader is referred to the Web version of this article.)

The major venom toxins selected for the study from the Viperidae family belonged to the phospholipase A_2_ (PLA_2_), snake venom metalloprotease (SVMP), and Kunitz-type serine protease (KSPI), protein families (Supplementary Table S1a). On the other hand, for the snakes belonging to the Elapidae family, the major toxins belonged to the PLA_2_ and three-finger toxin (3FTx) protein families (Supplementary Table S1b). Subsequently, after subjecting the selected toxins to multiple sequence alignment and selecting the most antigenic regions covering the active site/s, 17 custom peptides were derived (Supplementary Tables S1a-b). An example of the alignment of PLA_2_ of Viperidae and Elapidae is shown in Fig. 1. The alignment of rest of the toxin families is shown in Supplementary Fig. S1.The physicochemical properties of all the custom peptides are summarized in Table 2.

**Table 2:** Summary of the custom peptides against the major toxins and/or against partial/negligible immuno-recognized toxins of snakes belonging to Viperidae and Elapidae families and their physicochemical properties.

Protein Family	Custom Peptides (against Viperidae)	Peptide Id	No. of Residues	Molecular weight (g/mol)	Extinction coefficient (M^−1^cm^−1^)	Iso-electric point (pH)	Net charge at pH 7	Estimated solubility	Species
PLA_2_	PKDATDRCCFVHDCCYC	Pep 1	17	1979.28	1280	5.12	−1.2	Good water solubility.	*Daboia russelii & Echis carinatus*
	CENRICECDKAAAICFRQNLNTY	Pep 2	23	2679.05	1280	6.03	−0.3	Good water solubility	*Echis carinatus*
SVMP	AVIMAHELSHNLGMYHDGKNC	Pep 3	21	2340.67	1280	6.34	−0.8	Fairly water solubility	*Daboia russelii*
	AVAMAHEMGHNLGMDHDGGGCNC	Pep 4	23	2327.59	0	4.97	−2.8	Fairly water solubility	*Echis carinatus*
KSPI	CFLRPDFGRYGHPRPRFYYN	Pep 5	20	2561.88	3840	10.13	3	Good water solubility.	*Daboia russelii*
	CNLAPESGRCRGHLRRIYYN	Pep 6	20	2378.7	2560	9.87	3	Good water solubility.	
Snaclec	EEILVDIVVSENIGKMYKIWC	Pep 18	21	2481.93	6970	4.04	−2.1	Good water solubility.	*Echis carinatus*
	SECFVLEKQSVFRTWVATPC	Pep 19	20	2330.68	5690	6.13	−0.1	Fairly water solubility	
**Protein Family**	**Custom Peptides (against Elapidae)**	**Peptide Id**	**No. of Residues**	**Molecular weight (g/mol)**	**Extinction coefficient (M**^**−**^**^1^cm**^**−**^**^1^)**	**Iso-electric point (pH)**	**Net charge at pH 7**	**Estimated solubility**	**Species**
Phospholipase A_2_	CGRGGSGTPVDDLDRCCQVHDNC	Pep 7	23	2407.61	0	4.18	−2.2	Good water solubility	*Naja naja and Naja kaouthia*
	CAAAVCDCDRLAAICFAGAPYN	Pep 8	22	2218.56	1280	3.71	−1.3	Fairly water solubility.	
	CGAGGSGTPVDALDRCCYVHDNC	Pep 9	23	2313.54	1280	4.01	−2.2	Good water solubility.	*Bungarus caeruleus*
	CARFVCDCDRTAAICFAKAPYN	Pep 10	22	2438.84	1280	7.66	0.7	Good water solubility.	
	CYGAAGTCARIVCDCDRTAALC	Pep 11	22	2236.61	1280	5.72	−0.3	Fairly water solubility.	
Three finger toxins	CYTKTWCDGFCSSRGKRVDLGC	Pep 12	22	2485.85	6970	8.13	1.7	Good water solubility.	*Bungarus caeruleus, Naja naja and Naja kaouthia*
	LGCAATCPTVKTGVDIQCCSTDNC	Pep 13	24	2403.77	0	3.71	−1.3	Fairly water solubility.	
	CYKKWWSDHRGTIIERGCGC	Pep 14	20	2398.75	12660	8.41	1.9	Good water solubility.	
	GCAATCPIAENRDVIECCSTDKC	Pep 15	23	2402.74	0	3.93	−2.3	Good water solubility.	
	GKNLCYKMYMVATPVVPVKRGC	Pep 16	22	2458.05	2560	9.96	3.9	Fairly water solubility.	
	CPKNSLLVKYVCCNTDRCN	Pep 17	19	2173.57	1280	8.13	1.7	Good water solubility.	

### 3.2 Peptide-antibody interactions

**Fig. 2:**
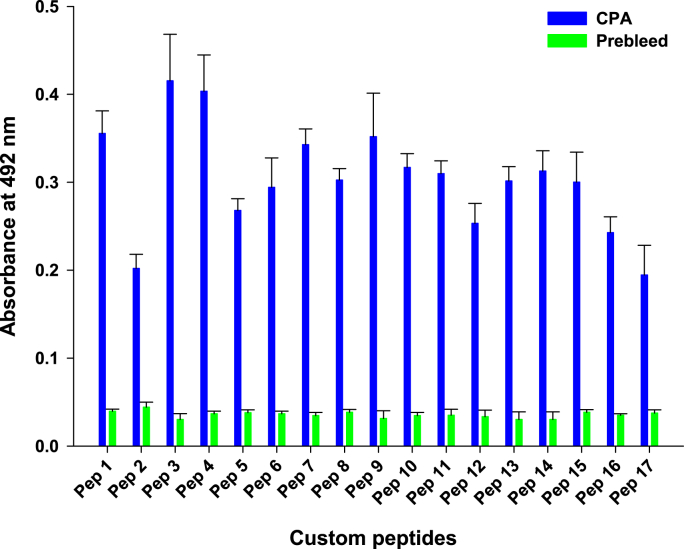
Determination of immune-recognition of custom peptides by respective purified CPAs using ELISA. 100 ng of each peptide was coated in triplicates in 96-well ELISA plate. The peptides were incubated with 200 ng of respective CPAs or prebleed serum (diluted to 1:5000) followed by recognition by HRP-conjugated anti-rabbit secondary antibody. The absorbance was recorded at 492 nm.

The rabbit antisera raised against the custom peptides exhibited virtuous titer values after the third booster dose, as determined by ELISA. For example, the titer values against the first five custom peptides (CPs) for the second and third booster doses are shown in Supplementary Fig. S2a. The dot blot analysis also exhibited good immunological cross-reactivity towards the toxin CPs (Supplementary Fig. S2b). Meanwhile, the pre-immune sera did not exhibit cross-reactivity towards the spotted CPs (Supplementary Fig. S2b). Moreover, the ELISA of purified CPAs against respective toxin CPs suggested appreciable immune-recognition (Fig. 2).

### 3.3 Formulation of custom peptide antibodies (CPA)

The purified CPAs raised against these toxins were mixed in different ratios to formulate two pools specific to Viperidae and Elapidae toxins based on the relative proportion of major toxins in the venom proteome of the ‘Big Four’ snakes of India (Table 1a).

### 3.4 CPAs show immuno-recognition of snake venom toxins against which they were raised

**Fig. 3:**
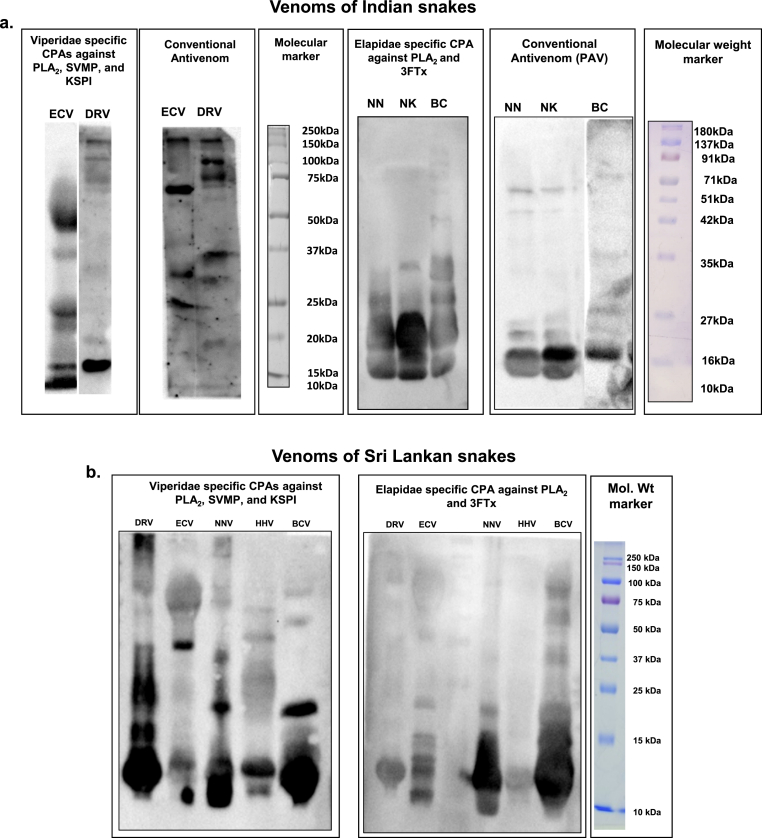
**(a)** Immunoblot assay to determine the immunological cross-reactivity between CPAs and crude venom from snakes of Indian origin [*Daboia russelii* venom (DRV), *Echis carinatus venom* (ECV), *Naja naja* venom (NNV), *Naja kaouthia* venom (NKV) and *Bungarus caeruleus* venom (BCV)]. **(b)** Immunoblot assay to determine the immunological cross-reactivity between CPAs and crude venom from snakes of Sri Lankan origin [*Daboia russelii* venom (DRV), *Echis carinatus venom* (ECV), *Hypnale hypnale* venom (HHV), *Naja naja* venom (NNV) and *Bungarus caeruleus* venom (BCV)]. The Viperidae-specific and Elapidae-specific CPAs (3 ng/μL) were used as primary antibodies. The blots were developed by using an ECL substrate.

The immuno-recognition capacity of the CPAs towards the venoms of ‘Big Four’ venomous snakes and *N. kaouthia* from India was assessed by immunoblot analysis. The Viperidae-specific and Elapidae-specific CPAs were evaluated separately to determine the ability of recognizing the venom toxins of all the snake venom samples. The immunoblot analysis depicts that the immuno-recognition of the targeted toxins (based on molecular range), especially the low molecular weight toxins by CPA, indicating polyclonal antibodies against custom peptides were raised successfully in rabbits (Fig. 3a and b, Supplementary Fig. S3a-b). The densitometry analysis showed that the extent of immuno-recognition of target toxins (against which antibodies were raised) was superior to the commercial PAV.

### 3.5 Neutralization of enzymatic and biological activities of venoms

The choice of biological activities for this study was based on those activities that remained poorly neutralized by commercial PAVs in our previous studies (Chanda et al., 2018, 2019; Dutta et al., 2017; Kalita et al., 2018b, 2018c; Mukherjee et al., 2016; Patra et al., 2017, 2019). A mild increase in the neutralization of SVMP and pro-coagulant activities were observed when the *D. russelli* and *E. carinatus* venoms were incubated with CPAs compared to commercial PAVs. In contrast, these activities were significantly neutralized when the FPAV was used (Table 3a). On the other hand, the PLA_2_ activity for all the tested venoms was significantly neutralized by the FPAV compared to commercial PAV (Table 3a, Table 3ba and 3b). Furthermore, the neutralization potency of anticoagulant activity of all tested Elapid venoms by FPAV was also more remarkable (p < 0.05) than PAV alone (Table 3b).

**Table 3a:** Neutralization of metalloprotease, phospholipase A_2,_ and pro-coagulant activities of crude *D. russelii* and *E. carinatus* venom (10 μg) by FPAV. Values are mean ± SD of triplicate determinations. ∗p < 0.05, the significance of the difference in neutralization potency between FPAV and PAV alone.

Antibody/Antivenom	Custom peptide designed against weak immunogenic major toxins	Venom: PAV/CPA/FPAV ratio (w/w)	% Neutralization		
			SVMP	PLA_2_ activity	Pro-coagulant activity
PAV	*D. russelli* venom	1:10	32.6 ± 0.8	7.1 ± 0.6	40.1 ± 2.3
CPAs		1:10	41.4 ± 2.3∗	19.4 ± 1.7∗	57.6 ± 3.9∗
FPVA		1:10	78.6 ± 3.4∗	47.1 ± 2.6∗	76.2 ± 3.7∗
PAV	*E. carinatus* venom	1:10	58.7 ± 2.1	6.8 ± 0.7	45.8 ± 2.4
CPAs		1:10	67.8 ± 1.3∗	16.7 ± 0.8∗	60.5 ± 1.3∗
FPVA		1:10	86.4 ± 1.4∗	42.7 ± 2.7∗	79.2 ± 1.7∗

**Table 3b:** Neutralization of phospholipase A_2_ and anticoagulant activities of crude *N. naja, N. kaouthia,* and *B. caeruleus* venom (10 μg) by formulated antivenom. Values are mean ± SD of triplicate determinations. ∗p < 0.05, the significance of the difference in neutralization potency between FPAV and PAV alone.

Antibody/Antivenom	Custom peptide designed against weak immunogenic major toxins	Venom: PAV/CPA/FPAV ratio (w/w)	% Neutralization	
			PLA_2_ activity	Anticoagulant activity
PAV	*N. naja* venom	1:10	3.8 ± 0.3∗	57.3 ± 1.7∗
CPAs		1:10	15.4 ± 1.1∗	68.5 ± 2.2∗
FPAV		1:10	68.4 ± 2.4∗	82.9 ± 3.1∗
PAV	*N. kaouthia* venom	1:10	3.4 ± 0.2∗	45.8 ± 3.4∗
CPAs		1:10	12.1 ± 1.4∗	61.2 ± 1.4∗
FPAV		1:10	57.1 ± 2.8∗	83.1 ± 2.8∗
PAV	*B. caeruleus* venom	1:10	1.8 ± 0.3∗	35.6 ± 1.7∗
CPAs		1:10	14.3 ± 2.1∗	51.5 ± 2.1∗
FPAV		1:10	66.7 ± 1.8∗	85.2 ± 3.4∗

### 3.6 The *in vivo* neutralization of lethality and pharmacological activities of venoms of the Big Four Indian snakes and *Naja kaouthia* by FPAV in a mouse model

The experimental mice in the study were injected with 5 LD_50_ doses of each snake venom. They were administered with different serially diluted doses of PAV (Supplementary Table S2a-e) to determine the challenge experiment dilution at which the PAV does not protect experimental mice. Subsequently, the pooled CPAs were mixed with diluted PAV to form FPAV. The challenge dose (5 LD_50_ value) of the ‘Big Four’ snake and *N. kaouthia* venoms were pre-incubated with PAV, FPAV, and normal saline (control) for 30 min at room temperature. After that, the mice (n = 6) were injected with the mixture, and the mice survived 24 h post injection. As mentioned, none of the mice are protected from death when treated with PAV or CPA alone (Table 4; Supplementary Tables S2a-e, S3a-e). Our studies, however, indicated that the protection was conferred when PAV was supplemented with CPA (FPAV) at different ratios for venom samples (Table 4; Supplementary Table S3a-e). The FPAVs could provide 50% boosted protection to mice injected with the venoms of *N. naja*, *N. kaouthia*, *D. russelii,* and *E. carinatus.* In contrast, the survivability rate was increased to 100% in the mice injected with the 5 lethal doses (5 LD_50_ value) of *B. caeruleus* venom when administered with the FPAVs (Table 4).

**Table 4:** Neutralization of lethality of the ‘Big Four’ and *N. kaouthia* venoms by diluted PAV, CPA and FPAV. The diluted PAV or the CPA did not protect mice against snake venom, whereas supplementation of CPA with the PAV significantly enhanced survivability (∗p < 0.05). The volume of buffer PBS, pH 7.4 was adjusted in the reaction mix to make the final injection volume of 500 μl. For each experiment, 6 mice were used, and the challenge venom dose was 5 LD_50_.

Snake venom	PAV		CPA		FPAV		
	Amount (mg)	Survivability	Amount (mg)	Survivability	CPA + PAV (in mg)	Ratio (CPA: PAV, w/w)	Survivability
*Naja naja*	2	0%	0.5	0%	0.5 + 2.0	1:4	50% ∗
*Daboia russelii*	1.5	0%	0.4	0%	0.4 + 1.5	1:3.75	50% ∗
*Bungarus caeruleus*	0.6	0%	0.3	16%	0.3 + 0.6	1:2	100% ∗
*Echis carinatus*	1.5	0%	0.18	0%	0.18 + 1.5	1:8.6	50% ∗
*Naja kaouthia*	35	0%	0.12	0%	0.12 + 35.0	1:300	50% ∗

In mice, the neutralization of venom-induced toxicities associated with the ‘Big Four’ snake venoms by PAVs, augmented with FPAV, was investigated. Various pharmacological activities, such as hemorrhagic (MHD), necrotizing (MND), pro-coagulant (MCP), defibrinogenating (MDD), and myotoxic activities (MMD), were assessed (Supplementary Table S4; Supplementary Fig. S4a-b). It was observed that these activities were effectively counteracted significantly more by FPAVs than PAV alone. However, their potencies varied, as detailed in Table 5, Supplementary Table S4, and Supplementary Fig. S4a-b.

**Table 5:** Neutralization of *in vivo* pharmacological activities of ‘Big Four’ snake venoms by PAV and FPAV. Values are means ± SD of five values. Neutralization of pharmacological activity is expressed as the activity of venom (mg) neutralized per mL of reconstituted (in 10 mL of water) PAV and FPAV (containing 1 mL of reconstituted PAV and different amounts of CPAs). ∗p < 0.05, the significance of the difference in neutralization potency between FPAV and PAV alone.

Venom	Neutralization of pharmacological properties	mg of venom neutralized per mL of PAV/FPAV				
		**Haemorrhagic activity (MHD**_**50**_**)**^a^	**Necrotizing activity (MND**_**50**_**)**^b^	**Coagulant activity (MCD-P**_**100**_**)**^c^	**Defibrinogenating activity (MDD**_**100**_**)**^d^	**Myotoxic activity (MMD**_**50**_**)**^e^
*Naja naja*	Neutralization by per mL of PAV	NA	NA	NA	NA	2.59 ± 0.59
	Neutralization by per mL of FPAV	NA	NA	NA	NA	3.24 ± 0.27∗
*Bungarus caeruleus*	Neutralization by per mL of PAV	NA	NA	NA	NA	3.17 ± 0.64
	Neutralization by per mL of FPAV	NA	NA	NA	NA	4.31 ± 0.33∗
*Daboia russelli*	Neutralization by per mL of PAV	13.64 ± 0.03	40.89 ± 0.49	NA	1.98 ± 0.32	2.38 ± 0.48
	Neutralization by per mL of FPAV	20.50 ± 0.03∗	61.51 ± 0.18∗	NA	3.17 ± 0.27∗	3.24 ± 0.27∗
*Echis carinatus*	Neutralization by per mL of PAV	1.20 ± 0.02	0.60 ± 0.01	9.11 ± 1.97	0.85 ± 0.14	2.97 ± 0.60
	Neutralization by per mL of FPAV	1.82 ± 0.06∗	0.91 ± 0.08∗	15.37 ± 0.35∗	1.37 ± 0.33∗	4.05 ± 0.27∗

NA indicates no activity by the venom.

a Minimum hemorrhagic dose (MHD) is defined as the amount of venom (in μg dry weight) which, when injected intradermally, induces in mice a 10 mm hemorrhagic lesion after a predefined time interval, usually 2–3 h, post injection.

b Minimum necrotizing dose (MND) is defined as the smallest amount of venom (in μg dry weight) which, when injected intradermally into anaesthetized mice, results in a necrotic lesion of 5 mm diameter post 3 days of treatment.

c The minimum coagulant dose on plasma (MCD-P) is the smallest amount of venom (in mg dry weight per liter of test solution or μg/mL) that induces clotting of citrated human plasma under the experimental conditions.

d Minimum defibrinogenating dose (MDD) is the minimum dose of venom that produces incoagulable blood in all mice within 1 h of intravenous injection.

e Minimum myotoxic dose (MMD) is characterized by the appearance of myoglobin in urine and by increments in the serum levels of muscle-derived enzymes, such as creatine kinase (CK).

## 4 Discussion

Over the past couple of decades application of proteomics and transcriptomics approach (venomics) has revolutionized our understanding of snake venom composition and variation (Chanda and Mukherjee, 2020; Kalita et al., 2018a; Patra and Mukherjee, 2020; Pla et al., 2019; Senji Laxme et al., 2021a, 2021b; Sintiprungrat et al., 2016; Tasoulis and Isbister, 2017). It has also been holistic in assessing anti-toxins in selectively cross-neutralizing venom toxin families (antivenomics) (Ledsgaard et al., 2018; Lomonte and Calvete, 2017) and provided better insight into the limitations of the current regime of snake venom treatment.

Recent years have witnessed the incorporation of innovative biotechniques to tackle limitations in conventional snakebite treatment strategies, such as natural and synthetic inhibitors, phage display techniques, nanoparticle engineering, and synthetic epitope methods. These approaches target enzymatic toxins, create antibody phage display libraries, and employ nanotechnology for precise toxin-neutralizing molecule delivery (Ferreira et al., 2006; Karain et al., 2016; Kini et al., 2018; Kulkeaw et al., 2009; O'Brien et al., 2018). Recent research has involved the development of a synthetic human antibody library aimed at identifying and creating an antibody capable of neutralizing the long-chain three-finger α-neurotoxins generated by medically significant snakes belonging to the Elapidae family (Khalek et al., 2024). Nonetheless, the cost-effectiveness of these emerging therapeutics for treating snake bites, often referred to as the “disease of poverty,” remains a significant concern.

In the present study, a catalogue of poorly or non-immunorecognized major toxins of the ‘Big Four’ venomous snakes and *N. kaouthia* venom from India and their counterparts from Sri Lanka and Pakistan was prepared based on the previous proteomic investigations of their venom proteome and the unbound fraction of the immuno-affinity column coupled with commercial antivenoms. These poorly or non-immunorecognized toxins comprise a significant proportion of their respective venom proteome. From Viperidae snake venoms, two custom peptides were designed from each of the PLA_2_, SVMP, and KSPI protein families, targeting their active sites. On the other hand, five peptides were designed from the PLA_2_, and six from 3FTx protein families for the Elapid snakes. Two of the PLA_2_ peptides were explicitly designed for the *Naja* genus, and the remaining three were particular to the *Bungarus* genus.

The titer values of the ELISA analysis indicated that the custom peptides could elicit robust immunogenicity in rabbits that remained stable across different booster doses. The dot-blot analysis suggests that the antisera from the rabbits raised against these custom peptides have depicted adequate antigen-antibody interactions. The immunoblot analysis by the purified CPAs displayed specificity in recognizing the targeted toxin epitopes against which they were designed. The CPAs have demonstrated pronounced immuno-recognition for the venoms of the ‘Big Four’ Indian snakes and *N. kaouthia*. Previously, it has been reported that though the low molecular weight toxins in the venoms play significant roles in venom-induced toxicity and adverse pharmacological effects, they are not adequately recognized by the PAVs (Chanda et al., 2019; Kalita et al., 2017; Patra et al., 2017, 2019; Tan et al., 2015b). However, in the current investigation, CPAs displayed greater immuno-recognition of low molecular weight toxins such as PLA_2_, KSPI, and 3FTx compared to commercial PAV, encouraging the development of better antivenoms against these toxins. These results of the *in vitro* assessment of the CPAs were vital before proceeding with the *in vivo* studies. Interestingly, CPAs show lower recognition of SVMPs, possibly because they target specific catalytic sites.

In contrast, polyvalent antivenoms (PAVs) contain antibodies against multiple epitopes of venom toxins, potentially including highly immunogenic sites rather than the enzymes' active sites. This difference may result in better overall toxin recognition but suboptimal neutralization, as the active sites of venom enzymes are not always the most immunogenic. A similar phenomenon has been noted with Russell's viper venom PLA_2_s (Kalita et al., 2017).

The efficacy of PAV supplemented with CPAs (FPAV) was analyzed through *in vivo* experiments. The challenge experiment determined the dosage at which PAVs could not protect experimental mice injected with five times the lethal dose of respective venoms. However, when FPAVs were used at this dosage in the challenge experiment, they were found to protect mice from venom-induced lethality. Since many low molecular mass poorly immunogenic toxins are not immune-recognized, PAVs alone do not neutralize their toxicity. However, this limitation of conventional PAV could be overcome when supplemented with CPAs containing antibodies against these toxins (FPAV).

In our study, the lethality induced by *B. caeruleus* venom was best neutralized by FPAVs rather than PAV. This effect may be attributed to the efficacy of CPAs raised against the most toxic β-bungarotoxin and long-chain neurotoxin. Envenomation doesn't always result in lethality; it also exhibits various pharmacological activities such as haemorrhage, necrosis, myotoxicity, and defibrinogenating activity (WHO, 2018). Ideally, for successful antivenom therapy, these toxic effects, sometimes challenging to mitigate along with lethality, should also be neutralized by the PAV, but this is hardly achieved (Faiz et al., 2017; Kularatne et al., 2009; S Girish and Kemparaju, 2011). Therefore, the WHO suggests that the efficacy of antivenom should be assessed not only by its ability to neutralize lethality but also by its capacity to neutralize venom-induced primary pharmacological activities (WHO, 2018). According to the current research, FPAVs can offer considerably better protection against venom-induced toxicities such as haemorrhage, necrosis, myotoxicity, and defibrinogenating activity than traditional PAVs. These findings indicated the potency of the FPAV and suggested they would be an effective alternate strategy in developing against the major and poorly immunogenic snake venom toxins.

Using CPAs offers several advantages; it is cost-effective, allows for cloning of the source immunogen peptide for large-scale production, and unlike conventional antivenom production, it is venom-independent. Additionally, supplementing conventional antivenom with CPAs (FPAV) can decrease the number of vials required for complete treatment, thus also reducing the PAV-induced adverse serum reactions.

This study is just a proof of concept regarding using custom peptides derived from poorly immunogenic snake venom toxins as an alternate strategy to enhance the efficacy of commercial antivenoms. Further studies and experiments regarding raising antibodies in horses, dose optimization, pharmacokinetics, and toxicokinetics are still warranted before the preclinical and clinical studies.

## 5 Conclusion

This work aims to review an adjuvant strategy that could enhance the perceived effectiveness of commercially available snake antivenoms in mitigating the negative impacts of low molecular weight toxins present in snake venoms.

To achieve this target, rabbits were injected with custom peptides to produce antibodies against specific peptides that were created using the low molecular weight venom toxins of the Elapidae and Viperidae families of snakes. Additionally, the combination of CPA and PAV resulted in FPAV providing excellent protection, 50% or more, to experimental mice at five times the lethal dose of the Indian snake venom under investigation. Before commercializing this method, much more detail must be considered, as this study is preliminary.

## Ethical statement

All animal experiments for antibody productions in rabbit were complied with the ARRIVE guidelines and approved by ethical requirements of the “Committee for Control and Supervision of Experiments in Animals (CPCSEA)” (BBI/IAEC/January/2019/02).

All animal experiments for *in vivo* neutralization were performed following the WHO recommendations and ARRIVE (Animal Research: Reporting of In Vivo Experiments) standards and the ethical requirements of the “Committee for Control and Supervision of Experiments in Animals (CPCSEA)" (R&D/ASVS-I/ED_50_-M/02/21)

## CRediT authorship contribution statement

**Abhishek Chanda:** Writing – review & editing, Writing – original draft, Validation, Methodology, Investigation, Formal analysis. **Nitin C. Salvi:** Writing – review & editing, Methodology, Investigation, Formal analysis. **Pravin V. Shelke:** Methodology, Investigation. **Bhargab Kalita:** Writing – review & editing, Writing – original draft, Validation, Methodology, Investigation, Formal analysis. **Aparup Patra:** Writing – review & editing, Writing – original draft, Methodology, Investigation, Formal analysis. **Upasana Puzari:** Writing – review & editing, Methodology, Investigation. **Milind V. Khadilkar:** Writing – review & editing, Methodology, Investigation, Formal analysis. **Ashis K. Mukherjee:** Writing – review & editing, Writing – original draft, Visualization, Validation, Supervision, Resources, Project administration, Methodology, Investigation, Funding acquisition, Formal analysis, Conceptualization.

## Declaration of competing interest

The authors declare that they have no known competing financial interests or personal relationships that could have appeared to influence the work reported in this paper.

## Data Availability

Data will be made available on request.
