# Supplementary material for: Supplementation of polyclonal antibodies, developed against epitope-string toxin-specific peptide immunogens, to commercial polyvalent antivenom, shows improved neutralization of Indian Big Four and Naja kaouthia snake venoms
Source: Toxicon X. 2024 Sep 26;24:100210. doi: 10.1016/j.toxcx.2024.100210 (PMC11471238; doi:10.1016/j.toxcx.2024.100210)

**Supplementary Information**

**Supplementation of polyclonal antibodies, developed against epitope-string toxin-specific peptide immunogens, to commercial polyvalent antivenom, shows improved neutralization of Indian Big Four and *Naja kaouthia* snake venoms**

**Abhishek Chanda^1,2^, Nitin C. Salvi^3^, Pravin V. Shelke^3^, Bhargab Kalita^1,4^, Aparup Patra^1,4^, Upasana Puzari^1^, M. V. Khadilkar^3^, Ashis K. Mukherjee^1,5^**

^1^MicrobialBiotechnology and Protein Research Laboratory, Department of Molecular Biology and Biotechnology, School of Sciences, Tezpur University, Tezpur- 784028, Assam, India

^2^Department of Pharmaceutical Sciences, College of Pharmacy, Oregon State University, Corvallis 97331, Oregon, USA.

^3^Premium Serums and Vaccines Pvt. Ltd, Narayangaon, Pune- 410504, Maharashtra, India

^4^Amrita Research Centre, Amrita VishwaVidyapeetham, Faridabad, Haryana, 121002, India

^5^Division of Life Sciences, Institute of Advanced Study in Science and Technology, Vigyan Path Garchuk, PaschimBoragaon, Guwahati 781035, Assam, India

**Supplementary Table S1a**: Designing of custom peptides against the major toxins and/or against partial/negligible immuno-recognised toxins of snakes belonging to Viperidae family.

| **Protein Family** | **Species** | **Accession No.** | **Protein Description** | **Origin** | **Sequence** | **# residues** | **Peptide Antigenic propensity** | **Site** |
| --- | --- | --- | --- | --- | --- | --- | --- | --- |
| Phospholipase A_2_ | *Daboia russelii*&*Echiscarrinatus* | P84674, B3RFI8, 1OZ6, P86368, P59071, A8CG86,A8CG78, Q6A3D6, P81458,1VIP,A8CG90 | Basic phospholipase A2 VRV-PL-V, phospholipase A2, Acidic phospholipase A2 DsM-a2/DsM-a2', Basic phospholipase A2 RVV-VD, Basic phospholipase A2 3, ammodytin I2(A) variant | SI*Echiscarrinatus, WI, EI and SI Daboia russelii* | PKDATDRCCFVHDCCY | 16 | 1.111 | Catalytic |
|  |  | P31100 | Acidic phospholipase A2 RV-7 | WI *Daboia russelii* | AQDATDRCCFVHDCCY | 16 | 1.117 | Catalytic |
|  |  | P84674, B3RFI8, 1OZ6, P86368, P59071, A8CG86,A8CG78, Q6A3D6, P81458,1VIP,A8CG90 | Basic phospholipase A2 VRV-PL-V, phospholipase A2, Acidic phospholipase A2 DsM-a2/DsM-a2', Basic phospholipase A2 RVV-VD, Basic phospholipase A2 3, ammodytin I2(A) variant | SI *Echiscarrinatus*, WI, EI and SI *Daboia russelii* | CENRICECDKAAAICFRRNLNTY | 23 | 1.0381 | Pharmacological |
|  |  |  |  |  | CENRICECDKAAAICFRQNLNTY | 23 | 1.0497 | Pharmacological |
|  |  |  |  |  | CENRICECDKAAAICFTKNLNTY | 23 | 1.0424 | Pharmacological |
| Kunitz-type serine protease | *Daboia russelii* | ABD24041.1 | Kunitz protease inhibitor-II [Daboia russelliirussellii] | EI | CFLRPDFGRYGHPRPRFYYN | 20 | 1.0432 | Active site |
|  |  | Q2ES49.1 | Kunitz-type serine protease inhibitor 2 | Pak | CFLRPDFGRYGHPRPRFYYN | 20 | 1.0432 | Active site |
|  |  | AFB74191.1 | protease inhibitor | Pak | CNLAPESGRCRGHLRRIYYN | 20 | 1.0479 | Active site |
|  |  | A8Y7P4.1 | Kunitz-type serine protease inhibitor B1 | WI | CNLAPESGRCRGHLRRIYYN | 20 | 1.0479 | Active site |
|  |  | ABD24040.1 | Kunitz protease inhibitor-I [Daboia russelliirussellii] | EI | CNLAPESGRCRGHLRRIYYN | 20 | 1.0479 | Active site |
|  |  | B8K1W0 | hemorrhagic metalloproteinase russelysin | *Wi, EI and SI Daboia russelii* | AVTMAHEIGHNLGLTHDGVYC | 21 | 1.0391 | Active site |
| Snake venom metalloprotease | *Daboia russelii* | K9JAW0 | factor X activator heavy chain | *Wi, EI and SI Daboia russelii*  SI Echiscarrinatus | AVIMAHELSHNLGMYHDGKNC | 21 | 1.0125 | Active site |
|  |  | AAB22477.1 | Coagulation factor X-activating enzyme beta-chain |  | AVIMAHELSHNLGMYHDGKNC | 21 | 1.0125 | Active site |
|  |  | E9JG47 | metalloproteinase |  | AVIMAHEMGHSLGMHHDSRSCNC | 23 | 1.0170 | Active site |
|  | *Echiscarrinatus* | E9JGG7 | metalloproteinase | SI Echiscarrinatus | AVAMAHEMGHNLGMDHDGNQCNC | 23 | 0.9808 | Active site |

**Supplementary Table S1b**: Designing of custom peptides against the major toxins and/or against partial/negligible immuno-recognised toxins of snakes belonging to Elapidae family.

| **Protein Family** | **Species** | **Accession No.** | **Protein Description** | **Origin** | **Sequence** | **# residues** | **Peptide Antigenic propensity** | **Site** |
| --- | --- | --- | --- | --- | --- | --- | --- | --- |
| Phospholipase A2 | *Naja naja and Naja kaouthia* | Q6T179, P15445,P00596, Q9I900 | phospholipase A2 isoform 4 precursor, Acidic phospholipase A2 2, Acidic phospholipase A2 1 | SI *Bungaruscaeruleu*s, WI and EI *Naja naja*, EI *Naja kaouthia* | CGRGGSGTPVDDLDRCCQVHDNC | 23 | 1.0381 | Catalytic |
|  |  | Q92084 | neutral phospholipase A2 | EI Naja kaouthia | CGRGGSGTPVDDLDRCCQIHDNC | 23 | 1.0276 | Catalytic |
|  |  | Q6T179 | phospholipase A2 isoform 4 precursor | SI *Bungaruscaeruleus*, WI *Naja naja*, | CAATVCDCDRLAAICFAGAPYN | 22 | 1.1200 | Pharmacological |
|  |  | P15445 | Acidic phospholipase A2 2 | WI and EI *Naja naja* | CAASVCDCDRLAAICFAGAPYN | 22 | 1.1250 | Pharmacological |
|  |  | P00596, Q9I900, Q92084 | Acidic phospholipase A2 1, Acidic phospholipase A2, neutral phospholipase A2 | EI *Naja naja* and *Naja kaouthia* | CAAAVCDCDRLAAICFAGAPYN | 22 | 1.1274 | Pharmacological |
|  | *SI Bungaruscaeruleus* | Q6SLM2 | Acidic phospholipase A2 1 | SI*Bungaruscaeruleus* | CGKGGSGTPVDDLDRCCYTHDHC | 23 | 1.0408 | Catalytic |
|  |  | Q9DF52 | BUNCE Basic phospholipase A2 KPA2 |  | CGKGGSGTPVDELDRCCYTHDNC | 23 | 1.0251 | Catalytic |
|  |  | Q8QFW4 | Basic phospholipase A2 beta-bungarotoxin A1 chain |  | CGAGGSGTPIDALDRCCYVHDNC | 23 | 1.0520 | Catalytic |
|  |  | Q8QFW3 | BUNCE Basic phospholipase A2 beta-bungarotoxin A2 chain |  | CGAGGSGTPVDALDRCCYVHDNC | 23 | 1.0625 | Catalytic |
|  |  | Q6SLM2 | Acidic phospholipase A2 1 |  | CARFVCDCDRTAAICFAKAPYN | 22 | 1.1060 | Pharmacological |
|  |  | Q9DF52 | BUNCE Basic phospholipase A2 KPA2 |  | CARFLCDCDRTAAICFASAPYN | 22 | 1.1036 | Pharmacological |
|  |  | Q8QFW4 | Basic phospholipase A2 beta-bungarotoxin A1 chain |  | CYGAAGTCARIVCDCDRTAALC | 22 | 1.0932 | Pharmacological |
|  |  | Q8QFW3 | BUNCE Basic phospholipase A2 beta-bungarotoxin A2 chain |  | CYGAAGTCGRIVCDCDRTAALC | 22 | 1.0842 | Pharmacological |
| Three finger toxins | *Naja naja, Naja kaouthia and SI Bungaruscaeruleus* | sp\|O42257 | Long neurotoxin 7 | SI *Bungaruscaeruleus* | CYTKTWCDGFCSSRGRRVELGC | 22 | 1.0354 | Active |
|  |  | sp\|P01391 | Alpha-Cobratoxin | EI *Naja naja and Naja kaouthia* | CYTKTWCDAFCSIRGKRVDLGC | 22 | 1.0546 | Active |
|  |  | sp\|P25669 | Long neurotoxin 2 | WI *Naja naja* | CYTKTWCDGFCSSRGKRVDLGC | 22 | 1.0389 | Active |
|  |  | sp\|P01427 | Short neurotoxin 1 | WI *Naja naja* | CYKKWWSDHRGTIIERGCGC | 20 | 1.0024 | Active |
|  |  | sp\|P59276 | Short neurotoxin II/ Cobrotoxin-c | EI *Naja naja* | CYKKWWSDHRGTIIERGCGC | 20 | 1.0024 | Active |
|  |  | sp\|P82463 | Muscarinic toxin-like protein 2 | WI *Naja naja* | GCAATCPIAENRDVIECCSTDKC | 23 | 1.0595 | Active |
|  |  | sp\|P01463 | Cytotoxin 2 | EI *Naja naja* | GKNLCYKMYMVATPMIPVKRGC | 22 | 1.0318 | Active |
|  |  | sp\|P60304 | Cytotoxin 1 | SI *Bungaruscaeruleus* | GKNLCYKMFMMSDLTIPVKRGC | 22 | 1.0102 | Active |
|  |  | sp\|P01440 | Cytotoxin 2 | WI *Naja naja* | GKNLCYKMYMVATPKVPVKRGC | 22 | 1.0478 | Active |
|  |  | sp\|Q9PST4 | Cytotoxin 2a | EI *Naja kaouthia* | GKNLCYKMYMVATPKVPVKRGC | 22 | 1.0478 | Active |
|  |  | sp\|P01463 | Cytotoxin 2 | EI *Naja naja* | CPKNSALVKYMCCNTDKCN | 19 | 1.0847 | Active |
|  |  | sp\|P60304 | Cytotoxin 1 | SI *Bungaruscaeruleus* | CPKNSLLVKYVCCNTDRCN | 19 | 1.1228 | Active |
|  |  | sp\|P01440 | Cytotoxin 2 | WI *Naja naja* | CPKSSLVLKYVCCNTDRCN | 19 | 1.1359 | Active |
|  |  | sp\|Q9PST4 | Cytotoxin 2a | EI *Naja kaouthia* | CPKSSLLVKYVCCNTDRCN | 19 | 1.1359 | Active |

**Supplementary Tables S2a-e** Serial dilution of polyvalent antivenom (PAV) to determine the challenge dose dilution (which does not protect experimental mice) when injected with 5 LD_50_ doses of each snake venom.

**Supplementary Table S2a.** Dose finding experiment for *Naja naja* venom (NnV). Number of mice used for each PAV dilution was 6.

| **S.No.** | **Venom (1 mg/mL) (µL)** | **(ASVS B.NoLyo 014) (µL)** | **Normal Saline (µL)** | **Death** |
| --- | --- | --- | --- | --- |
| 1 | 50 | 50 (Undiluted) | 400 | 0 |
| 2 | 50 | 100 (Undiluted) | 300 | 0 |
| 3 | 50 | 50 (1:1.5)^‡^ | 400 | 6 |
| 4 | 50 | 50 (1:2.25) | 400 | 6 |
| 5 | 50 | Nil | 450 | 6 |

^‡^Protection by undiluted PAV, but the death of mice was observed at 1:1.5 dilution of PAV. Hence, this dilution was considered for challenge experiments using CPAs.

**Supplementary Table S2b.** Dose finding experiment for *Daboia russelii* venom (DRV). Number of mice used for each PAV dilution was 6.

| **S.No.** | **Venom (1 mg/mL) (µL)** | **(ASVS B.NoLyo 014) (µL)** | **Normal Saline (µL)** | **Death** |
| --- | --- | --- | --- | --- |
| 1 | 50 | 50 (Undiluted) | 400 | 0 |
| 2 | 50 | 100 (Undiluted) | 300 | 0 |
| 3 | 50 | 50 (1:1.5) | 400 | 2 |
| **4** | **50** | **50 (1:2.25)** | **400** | **5** |
| 5 | 50 | Nil | 450 | 6 |

^‡^Protection by undiluted serum, but the death of mice was observed at 1:2.25 dilution of PAV. Hence, this dilution was considered for challenging experiments using CPAs.

**Supplementary Table S2c.** Dose finding experiment for *Bungaruscaeruleus* Venom (BCV). Number of mice used for each PAV dilution was 6.

| **S. No.** | **Venom (0.3 mg/mL) (µL)** | **(ASVS B.NoLyo 014) (µL)** | **Normal Saline (µL)** | **Death** |
| --- | --- | --- | --- | --- |
| 1 | 50 | 50 (1:1.5) | 400 | 0 |
| 2 | 50 | 50 (1:2.25) | 400 | 0 |
| 3 | 50 | 50 (1:3.37) | 400 | 0 |
| 4 | 50 | 50 (1:5.06) | 400 | 0 |
| 5 | 50 | 50 (1:7.59)^‡^ | 400 | 6 |
| 6 | 50 | Nil | 450 | 6 |

^‡^Protection by undiluted serum, but mice death was observed at 1:7.59 dilution of PAV. Hence, this dilution was considered for challenging experiments using CPAs.

**Supplementary Table S2d.** Dose finding experiment for *Echiscarinatus* venom (ECV). Number of mice used for each PAV dilution was 6.

| **S.**  **No.** | **Venom (1.5 mg/mL) (µL)** | **(ASVS B.NoLyo 014) (µL)** | **Normal Saline (µL)** | **Death** |
| --- | --- | --- | --- | --- |
| 1 | 50 | 50 (Undiluted) | 400 | 0 |
| 2 | 50 | 100 (Undiluted) | 300 | 0 |
| 3 | 50 | 50 (1:1.5) | 400 | 3 |
| **4** | **50** | **50 (1:2.25)** | **400** | **6** |
| 5 | 50 | Nil | 450 | 6 |

^‡^Protection was achieved by undiluted serum, but death was observed at 1:2.25 dilution of PAV; hence, this dilution was considered for challenge experiments using CPAs.

**Supplementary Table S2e.** Dose finding experiment for *Naja kouthia* venom (NkV). Number of mice used for each PAV dilution was 6.

| **S.No.** | **Venom (3.4 mg/mL) (µL)** | **(ASVS B.NoLyo 014) (µL)** | **Normal Saline (µL)** | **Death** |
| --- | --- | --- | --- | --- |
| 1 | 50 | 200 (Undiluted) | 250 | 6 |
| 2 | 50 | 250 (Undiluted) | 200 | 6 |
| 3 | 50 | 300 (Undiluted) | 150 | 6 |
| 4 | 50 | 350 (Undiluted)^‡^ | 100 | 6 |
| 5 | 50 | 400 (Undiluted) | 50 | 0 |
| 6 | 50 | Nil | 450 | 6 |

^‡^Protection by undiluted serum; however, at 400 µL, and as all lower volume proportions show death of mice (or no protection), 350 µL (Undiluted) volume of antivenom is considered for challenge experiments using CPAs.

**Supplementary Tables S3a-e** Dose finding assay for custom peptide antibodies (CPA) when supplemented with PAV at different ratios

**Supplementary Table S3a.** Dose finding experiment for *Naja naja* venom. Number of mice used for each concentration of CPA was 6

| **Venom (1mg/mL) µL** | **PAV (100 mg/mL) µL** | **CPA (1.2 mg/mL) µL** | **Normal saline (µL)** | **Total volume (µL)** | **Death** | **% Protection** |
| --- | --- | --- | --- | --- | --- | --- |
| 50 (5 LD50, Challenge Dose) | Nil | Nil | 450 | 500 | 6 | 0.0 |
| 50 | 50 (1:1.5) | - | 450 | 500 | 5 | 16.7 |
| 50 | 50 (1:1.5) | 250 | 200 | 500 | 5 | 16.7 |
| 50 | 50 (1:1.5) | 300 | 150 | 500 | 5 | 16.7 |
| 50 | 50 (1:1.5) | 350 | 100 | 500 | 5 | 16.7 |
| 50 | 50 (1:1.5) | 400 | 50 | 500 | 3 | 50.0 |

**Supplementary Table S3b.** Dose finding experiment for *Daboia russelii* venom. Number of mice used for each concentration of CPA was 6

| **Venom (1mg/mL) µL** | **PAV (100 mg/mL) µL** | **CPA (1.2 mg/mL) µL** | **Normal saline (µL)** | **Total volume (µL)** | **Death** | **% Protection** |
| --- | --- | --- | --- | --- | --- | --- |
| 50 (5 LD50, Challenge Dose) | Nil | Nil | 450 | 500 | 6 | 0.0 |
| 50 | 50 (1:2.25) | Nil | 450 | 500 | 6 | 0.0 |
| 50 | 50 (1:2.25) | 250 | 200 | 500 | 5 | 16.7 |
| 50 | 50 (1:2.25) | 300 | 150 | 500 | 5 | 16.7 |
| 50 | 50 (1:2.25) | 350 | 100 | 500 | 3 | 50.0 |
| 50 | 50 (1:2.25) | 400 | 50 | 500 | 3 | 50.0 |

**Supplementary Table S3c.** Dose finding experiment for *Bungaruscaeruleus* venom. Number of mice used for each concentration of CPA was 6

| **Venom (0.3 mg/mL) µL** | **PAV (100 mg/mL) µL** | **CPA (0.9 mg/mL) µL** | **Normal saline (µL)** | **Total volume (µL)** | **Death** | **% Protection** |
| --- | --- | --- | --- | --- | --- | --- |
| 50 (5 LD50, Challenge Dose) | Nil | Nil | 450 | 500 | 6 | 0.0 |
| 50 | 50 (1:7.59) | Nil | 450 | 500 | 6 | 0.0 |
| 50 | 50 (1:7.59) | 250 | 200 | 500 | 3 | 50.0 |
| 50 | 50 (1:7.59) | 300 | 150 | 500 | 2 | 66.7 |
| 50 | 50 (1:7.59) | 350 | 100 | 500 | 0 | 100.0 |
| 50 | 50 (1:7.59) | 400 | 50 | 500 | 0 | 100.0 |

**Supplementary Table S3d.** Dose finding experiment for *Echiscarinatus* venom. Number of mice used for each concentration of CPA was 6

| **Venom (1.5 mg/mL) µL** | **PAV (100 mg/mL) µL** | **CPA (0.45 mg/mL) µL** | **Normal saline (µL)** | **Total volume (µL)** | **Death** | **% Protection** |
| --- | --- | --- | --- | --- | --- | --- |
| 50 (5 LD50, Challenge Dose) | Nil | Nil | 450 | 500 | 6 | 0.0 |
| 50 | 50 (1:2.25) | Nil | 450 | 500 | 6 | 0.0 |
| 50 | 50 (1:2.25) | 250 | 200 | 500 | 6 | 0.0 |
| 50 | 50 (1:2.25) | 300 | 150 | 500 | 5 | 16.7 |
| 50 | 50 (1:2.25) | 350 | 100 | 500 | 5 | 16.7 |
| 50 | 50 (1:2.25) | 400 | 50 | 500 | 3 | 50.0 |

**Supplementary Table S3e.** Dose finding experiment for *Naja kaouthia* venom. Number of mice used for each concentration of CPA was 6

| **Venom (3.4 mg/mL) µL** | **PAV (100 mg/mL) µL** | **CPA (100 µL)^a^** | **Normal saline (µL)** | **Total volume (µL)** | **Death** | **% Protection** |
| --- | --- | --- | --- | --- | --- | --- |
| 50 (5 LD50, Challenge Dose) | Nil | Nil | 450 | 500 | 6 | 0.0 |
| 50 | 350 (undiluted) | Nil | 100 | 500 | 6 | 0.0 |
| 50 | 350 (undiluted) | 1.2 mg/mL | 0 | 500 | 6 | 0.0 |
| 50 | 350 (undiluted) | 1.8 mg/mL | 0 | 500 | 6 | 0.0 |
| 50 | 350 (undiluted) | 2.4 mg/mL | 0 | 500 | 6 | 0.0 |
| 50 | 350 (undiluted) | 3.0 mg/mL | 0 | 500 | 6 | 0.0 |
| 50 | 350 (undiluted) | 4.0 mg/mL | 0 | 500 | 6 | 0.0 |
| 50 | 350 (undiluted) | 5.0 mg/mL | 0 | 500 | 3 | 50.0 |

^a^ The undiluted PAV up to 350 µL have no protection against venom-induced lethality. The maximum volume to be injected into the mice was 500 µL. Therefore, a fix volume (100 µL) of increasing CPA concentration (1.2-5.0 mg/mL) was used for neutralization assay.

**Supplementary Table S4:**The in vivo pharmacological activities of ‘Big Four’ snake venom was determined in the mice model (n=6). The detailed methodologies are mentioned in the text.

| **Venom** | **Pharmacological activities in mice (n=6)** | | | | |
| --- | --- | --- | --- | --- | --- |
|  | **MHD ^a^(µg/mouse)** | **MND ^b^(µg/mouse)** | **MCD-P ^c^(µg/mL)** | **MDD ^d^(µg/mL)** | **MMD ^e^(µg/mouse)** |
| *Naja naja* | NO MHD | NO MND | NO MCD-P | NO MDD | 12.00 + 0.71 |
| *Bungaruscaeruleus* | NO MHD | NO MND | NO MCD-P | NO MDD | 16.00 + 0.71 |
| *Daboia russelli* | 5.00 + 0.16 | 60.00 + 0.45 | NO MCD-P | 6.20 + 0.45 | 12.00 + 0.71 |
| *Echiscarinatus* | 1.00 + 0.04 | 2.00 + 0.05 | 15.00 + 0.71 | 0.79 + 0.04 | 10.00 + 1.00 |

^a^ Minimum hemorrhagic dose (MHD) is defined as the amount of venom (in μg dry weight) which, when injected intradermally, induces in mice a 10 mm hemorrhagic lesion after a predefined time interval, usually 2–3 h, post injection.

^b^ Minimum necrotizing dose (MND) is the smallest amount of venom (in μg dry weight), which when injected intradermally into anaesthetized mice, results in a necrotic lesion of 5 mm diameter post three days of treatment.

^c^ The minimum coagulant dose on plasma (MCD-P) is the smallest amount of venom (in mg dry weight per liter of test solution or μg/mL) that induces clotting of citrated human plasma under the experimental conditions.

^d^ Minimum defibrinogenating dose (MDD) is the minimum dose of venom that produces incoagulable blood in all mice within one hour of intravenous injection.

^e^Mimimummyotoxic dose (MMD) is characterized by the appearance of myoglobin in urine and by increments in the serum levels of muscle-derived enzymes, such as creatine kinase (CK).

**Supplementary Fig S1**.Multiple sequence alignments of KSPI, SVMP, and 3FTx of Viperidae and Elapidae showing the selected peptides (in the box). The conserved regions are marked in green, and the active sites are marked in red.

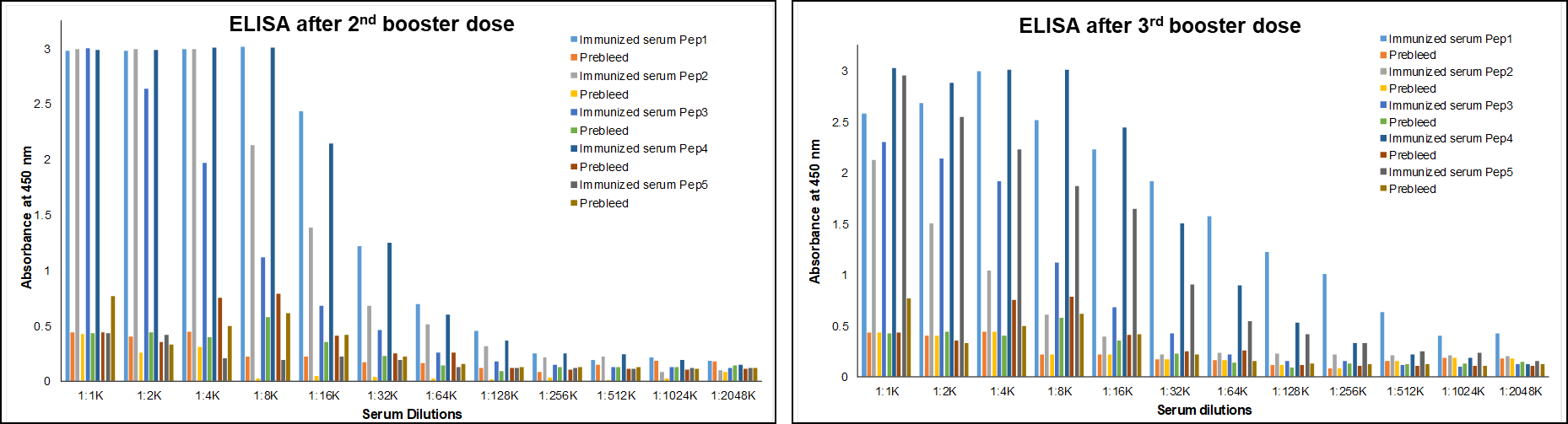
**Supplementary Fig S2a**. Determination of antigen-antibody titerafter 2nd and 3rd booster dose by ELISA. The absorbance was recorded at 450 nm

**Supplementary Fig S2b**. Dot blot assay determined immunological cross-reactivity between custom peptides and corresponding polyclonal antibodies raised in rabbits

**Supplementary Fig S3:** Full length un-edited blot image of **a.** Figure 3a **b.** figure 3b


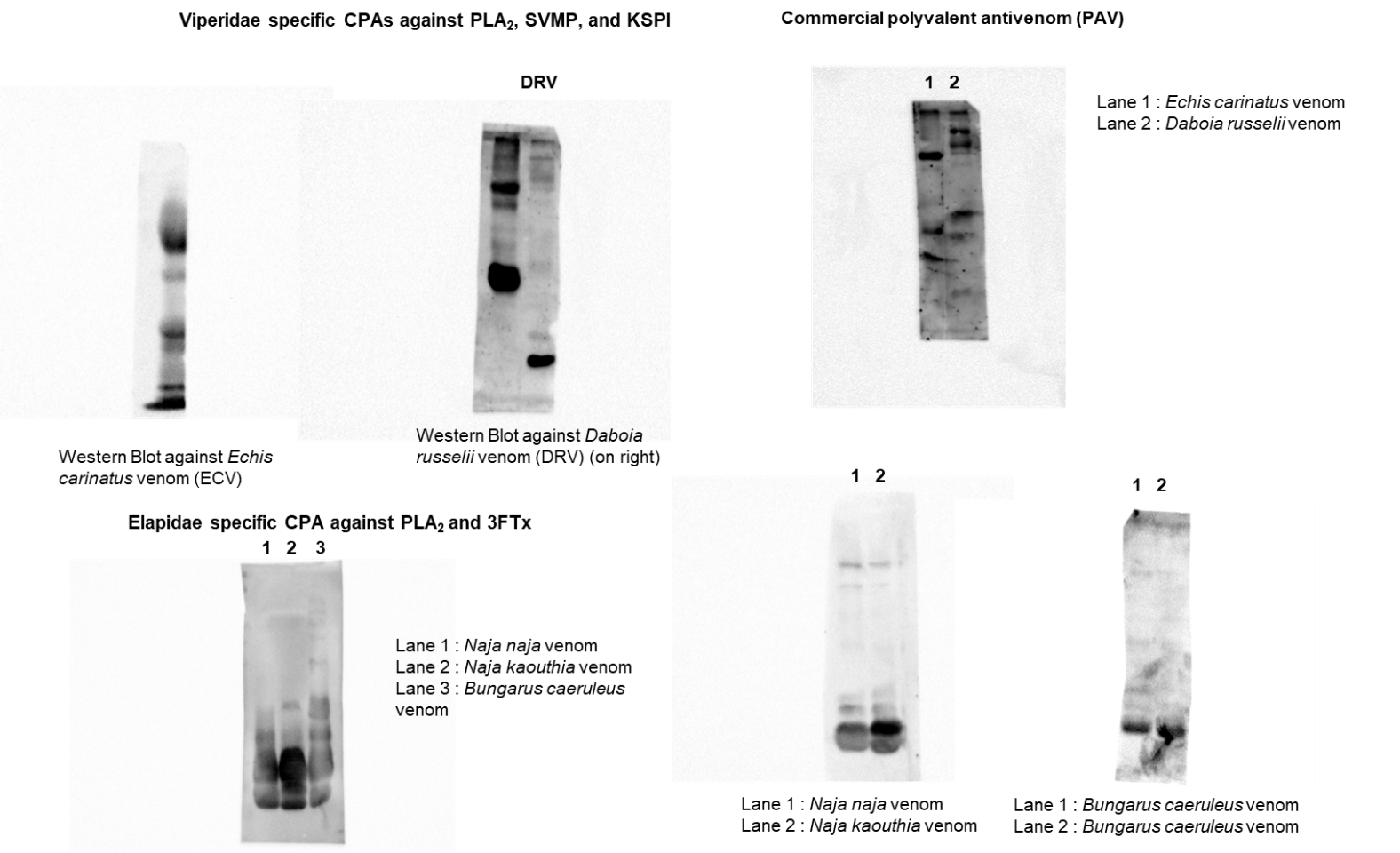


**a**

**
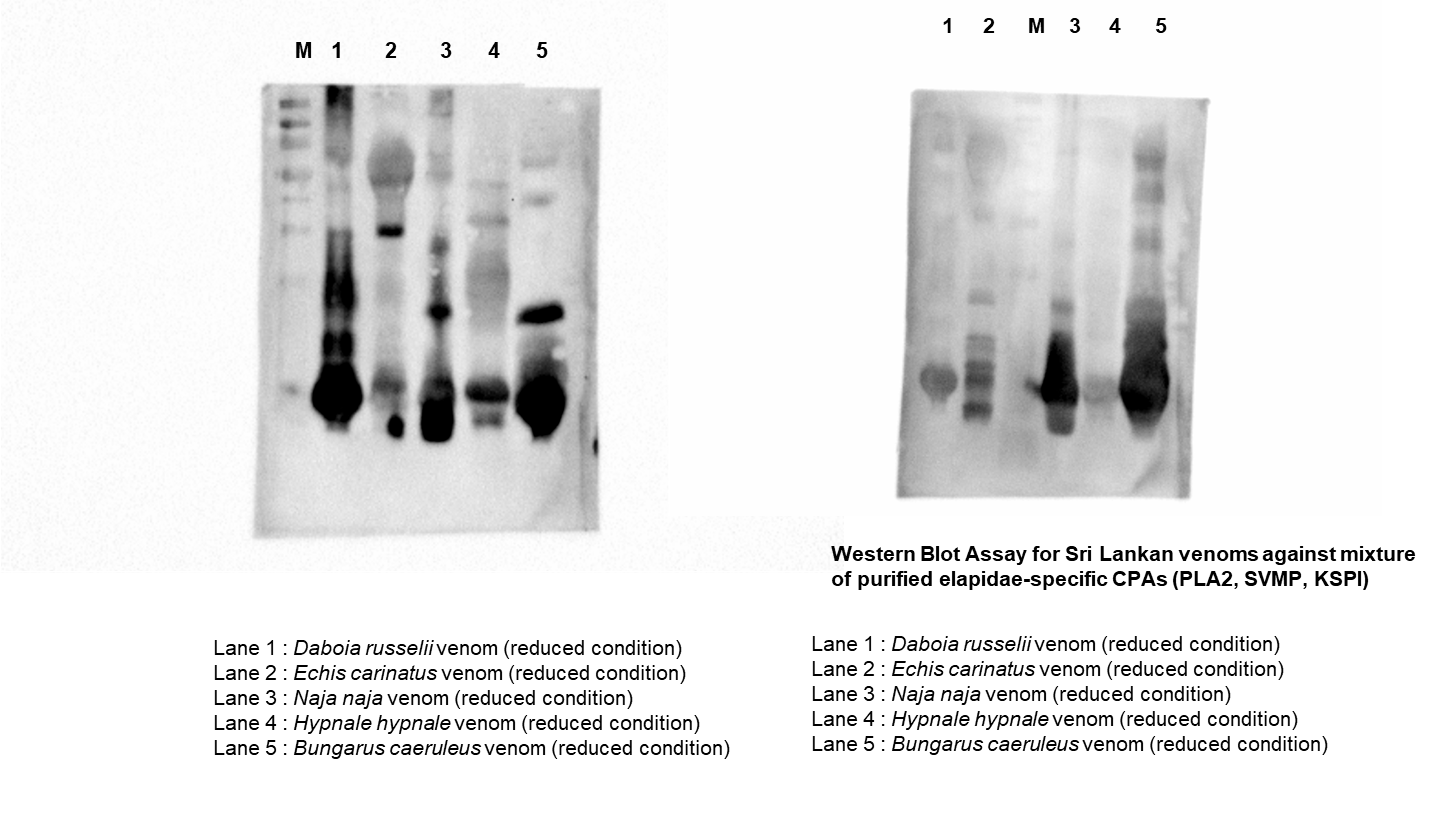
**

**b**

**Supplementary Fig S4:** Photograph of in vivo pharmacological activities of ‘Big four’ snake venoms in mice(n=6) **a**. Elapidae snakes, **b.** Viperidae snakes


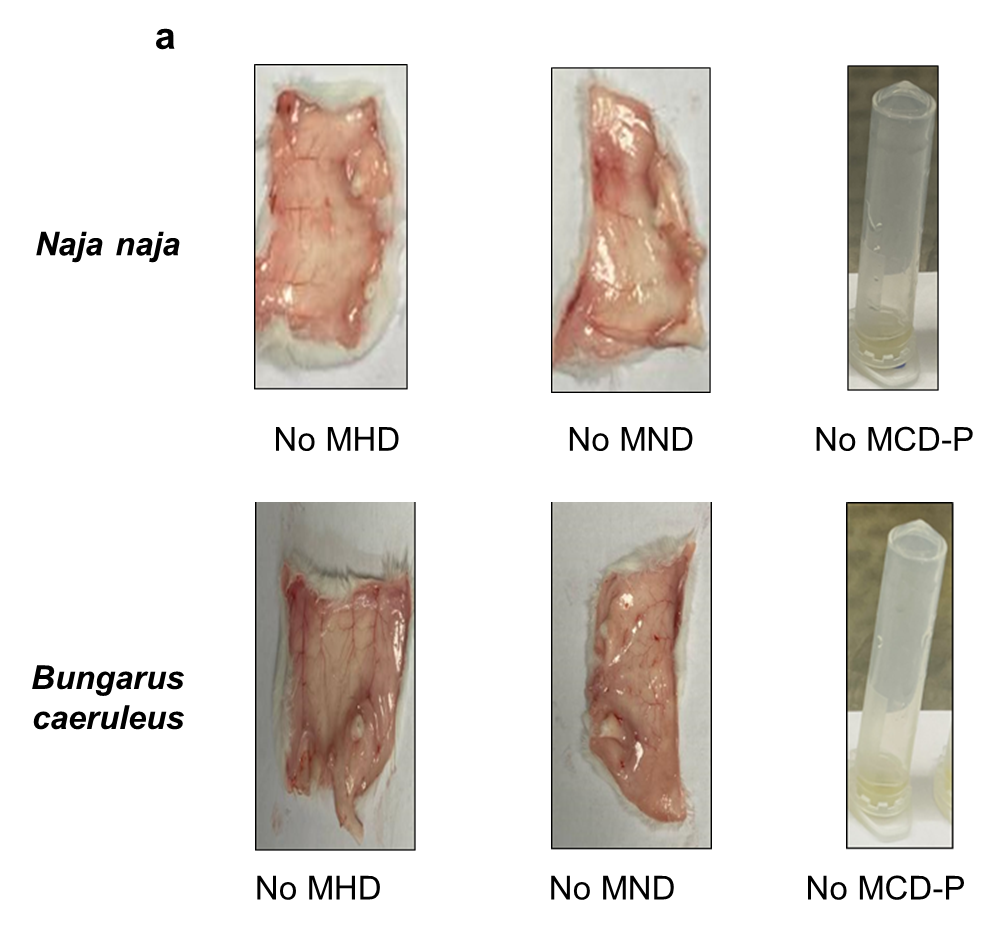


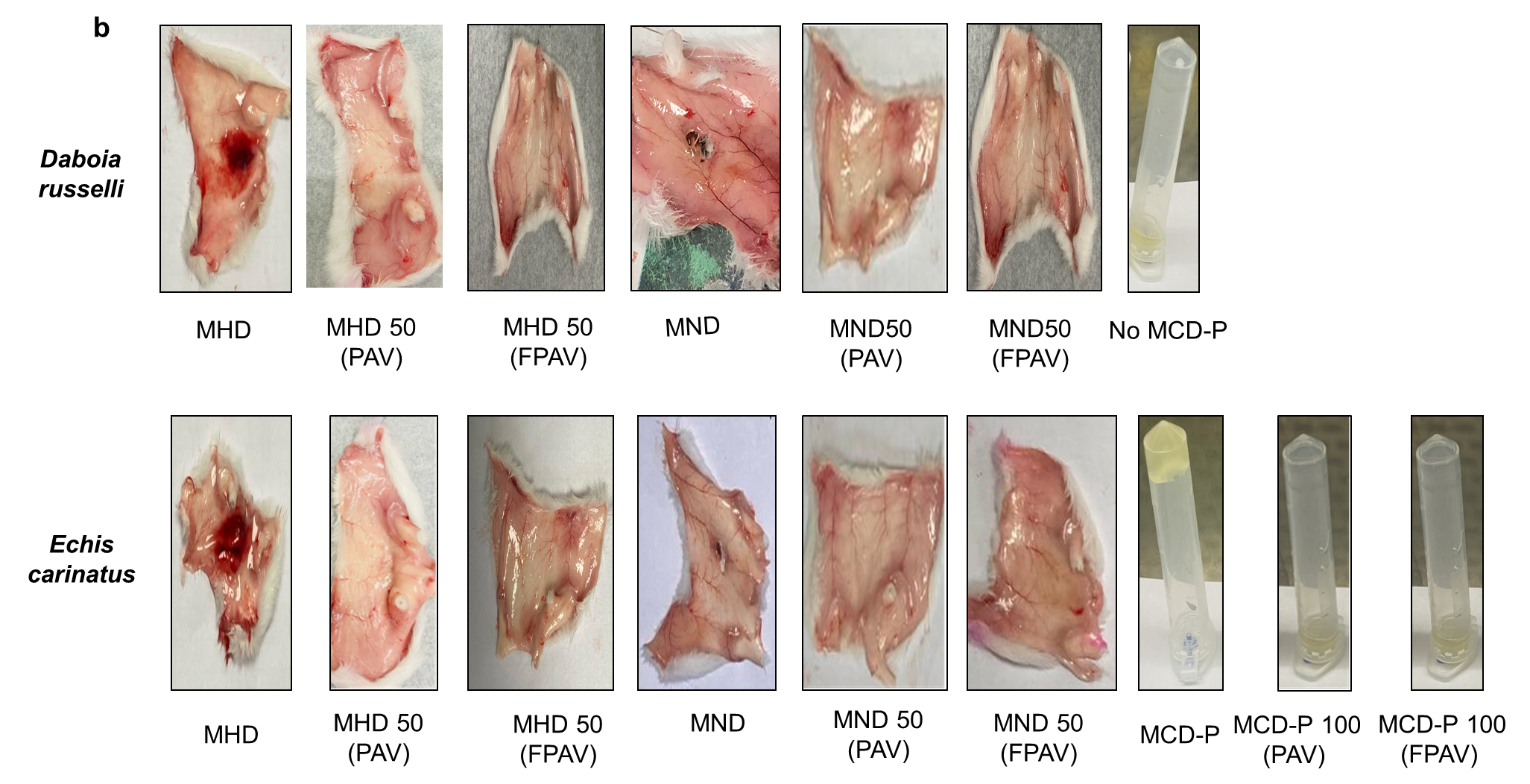

Supplement: Multimedia component 1 [file mmc1.docx]
